# Concern regarding H3-subtype avian influenza virus

**DOI:** 10.3389/fmicb.2023.1327470

**Published:** 2023-12-07

**Authors:** Jiantao Yu, Qiucheng Yao, Jing Liu, Yan Zhou, Miaotong Huo, Ye Ge

**Affiliations:** College of Coastal Agricultural Sciences, Guangdong Ocean University, Zhanjiang, China

**Keywords:** avian influenza virus, H3-subtype, genetics, evolution, interspecies transmission

## Abstract

The H3-subtype of avian influenza virus (AIV) is one of the most frequently detected low pathogenic avian influenza virus (LPAIV) subtypes in birds and fowls, causing substantial economic loss to the poultry industry. Most importantly, besides poultry, mammals could also be infected with it, such as swines, canines, equines, felines, and humans, posing a serious public health threat. This allows the virus to persist widely in poultry and wild birds for a long time, where it may mix with other subtypes, providing conditions for viral recombination or reassortment. Currently, the monitoring of H3-subtype AIV is inadequate, and there is a lack of effective prevention and control measures for H3-subtype AIV. Here, the epidemiology, phylogeny, and genetic variation of H3-subtype AIV were analyzed, and nonsynonymous and synonymous substitution rates (dN/dS) were calculated. Through these steps, we aimed to clarify the current epidemiological feature and evolutionary characteristics of H3-subtype AIV, and provide an operative reference for future scientific control of H3-subtype AIV.

## 1 Introduction

Avian influenza virus (AIV) is a type A influenza virus and one of the most significant zoonotic diseases that poses a severe threat to both the poultry industry and human health worldwide (Wei-Wen Hsiao et al., 2023; Yang et al., 2023). The AIV genome consists of eight segments of single-stranded negative-sense RNA. Haemagglutinin (HA) and neuraminidase (NA), encoded by segments 4 and 6, respectively, are the main antigenic glycoproteins on the surface of AIV (Jiang et al., 2023; Yang et al., 2023). According to the antigenic differences between HA and NA on its surface, AIV were divided to 18 HA subtypes and 11 NA subtypes (Liu et al., 2019). Except for H17N10 and H18N11, which were isolated from bats (Yang et al., 2021), all other subtypes of influenza A viruses had been identified in wild birds (Kawaoka et al., 1990; RÖHM et al., 1996; Liu et al., 2005; Wang et al., 2014). Different subtypes of AIV have different degrees of pathogenicity to poultry and mainly divided in highly pathogenic avian influenza (HPAI) and lowly pathogenic avian influenza (LPAI). The former is invoked by some H5 and H7 subtype virus strains, while other AIVs usually show low pathogenicity to poultry.

The H3-subtype AIV exists widely in birds, humans, and other mammals. Although its pathogenicity is low, it has many subtype combinations, a wide distribution and complex biological characteristics (Wang et al., 2014). To date, researchers have discovered a total of nine subtypes of H3NX AIV, with H3N2 and H3N8 as the most frequently detected subtypes in birds and canines. And swine and feline influenza were primarily caused by the H3N2 subtype of the influenza A virus, while equine influenza was mainly caused by the H3N8 influenza A virus. In addition, because of the low pathogenicity of H3-subtype AIV, infected animals generally show no symptoms or mild clinical symptoms, so the impact on the breeding industry and human health is often overlooked, which allows H3-subtype AIV to continue to spread and evolve in nature.

H3-subtype AIV has a higher isolation rate in wild birds than in mammals (including humans), especially in wild waterfowl, which are considered its natural hosts. Domestic ducks are considered an important intermediate host for AIV transmission from wild waterfowl to terrestrial fowl and one crucial determinant influencing the genetic diversity of AIV (Huang et al., 2010, 2012; Kim et al., 2012). Avian influenza surveillance data indicate that H3- subtypes of AIV were prevalent in domestic ducks and could be mixed within them, providing good conditions for new subtypes and mutations in genes affecting virulence via antigenic transformation. One report showed that H3N8 in wild birds possessed dual-receptor binding properties in the HA protein, which supports the occurrence of this type of AIV interspecies transmission (Tian et al., 2023). Notably, studies had shown that H3NX AIV has the ability to provide genetic fragments for reassortment in other subtypes of influenza viruses, leading to the production of new highly pathogenic strains and thus to influenza outbreaks (Peng et al., 2013; Wenqiang et al., 2017; Zhao et al., 2023). The H3-subtype AIV can also undergo recombination to alter its pathogenicity and has the potential to break through the interspecific barrier to infect humans (Song et al., 2008). Studies have shown that H3N2 influenza in Hong Kong in 1968 was also due to recombination with the human H2N2 virus NA, PB2, PA, NP, M and NS genes, but the population does not have immunity to the new HA and NA, resulting in the rapid spread of the new virus, causing a human flu pandemic (Lindstrom et al., 2004). In April 2022, China notified the WHO of the world’s first known human infection with the H3N8 subtype of avian influenza virus (Zhu et al., 2023).

## 2 Epidemiological features of the AIVs H3-subtype

### 2.1 Geographic arrangement of the H3-subtype

**Figure 1:**
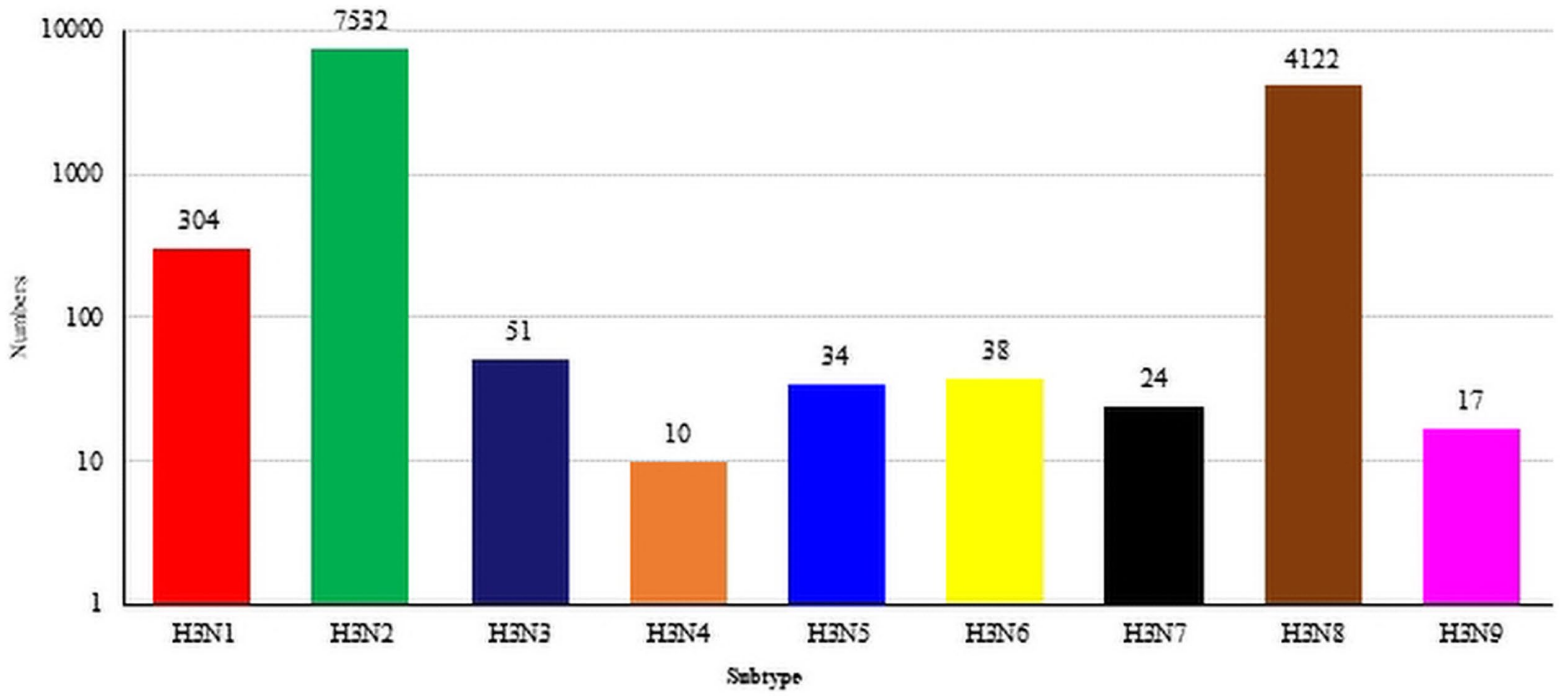
The global distribution of H3NX. The HA sequences were downloaded from the GISAID and NCBI databases; H3N? (unidentified NA subtype) sequences are not included here.

As of July 2023, the GISAID and NCBI databases included sum total of 12,134 H3-subtype AIV HA sequences, which included 304 H3N1 sequences, 7,532 H3N2 sequences, 51 H3N3 sequences, 10 H3N4 sequences, 34 H3N5 sequences, 38 H3N6 sequences, 24 H3N7 sequences, 4,122 N8 sequences, and 17 H3N9 sequences (Figure 1).

**Figure 2:**
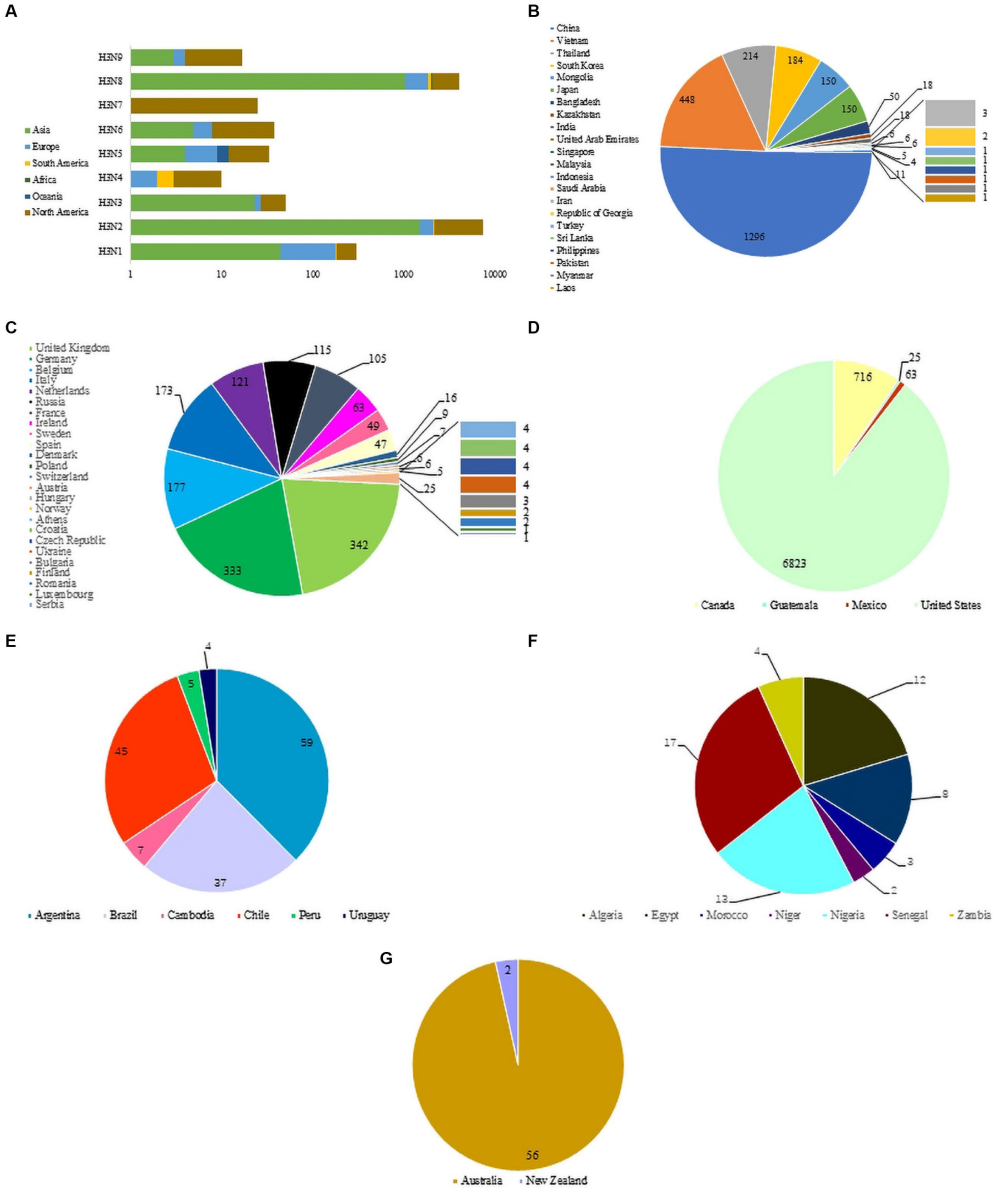
**(A)** Distribution of H3 AIVs on different continents. **(B)** Asian circulation of H3 virus. **(C)** North American circulation of H3 virus. **(D)** European circulation of H3 virus. **(E)** South American circulation of H3 virus. **(F)** African circulation of H3 virus. **(G)** Oceanian circulation of H3 virus.

The nine NA subtypes of H3NX were mainly distributed in North America, Asia and Europe. North America includes all 9 H3-subtypes AIV (H3N1-H3N9), Asia and Europe are dominated by H3N1, H3N2, H3N6 and H3N8, South America and Africa are dominated by H3N2 and H3N8, and Oceania includes H3N1, H3N2, H3N5 and H3N8 (Figure 2A). H3-subtype AIV is distributed in all major areas except Antarctica. The Eurasian continent has the widest distribution of H3-subtype AIV (the transmission range included 22 Asian countries and 25 European countries). The country with the highest detection rate of H3-subtype AIV in Asia was China (1,296 strains), followed by Vietnam, Thailand, South Korea, Mongolia and Japan (Figure 2B). The country with the highest detection of H3-subtype AIV in Europe was the United Kingdom, which included 342 strains, followed by Germany, Belgium, Italy, the Netherlands, Russia, France and others (Figure 2C). The United States (6,823 strains) had the highest detection rate of H3-subtype AIV in North America, followed by Canada, Mexico and Guatemala (Figure 2D). H3-subtype AIV was also detected in South America, but the detection rate was low; Argentina had 59 strains, followed by Chile, Brazil, Colombia, Peru and Uruguay (Figure 2E). H3-subtype AIV detection in African countries was lower than that in other places, including Senegal (17 strains), Nigeria (13 strains), Algeria (12 strains), Egypt (8 strains), Zambia (4 strains), Morocco (3 strains) and Niger (2 strains) (Figure 2F). In Oceania, H3-subtype AIV was detected only in Australia and New Zealand (Figure 2G).

### 2.2 Epidemic of H3-subtype avian influenza in poultry

**Figure 3:**
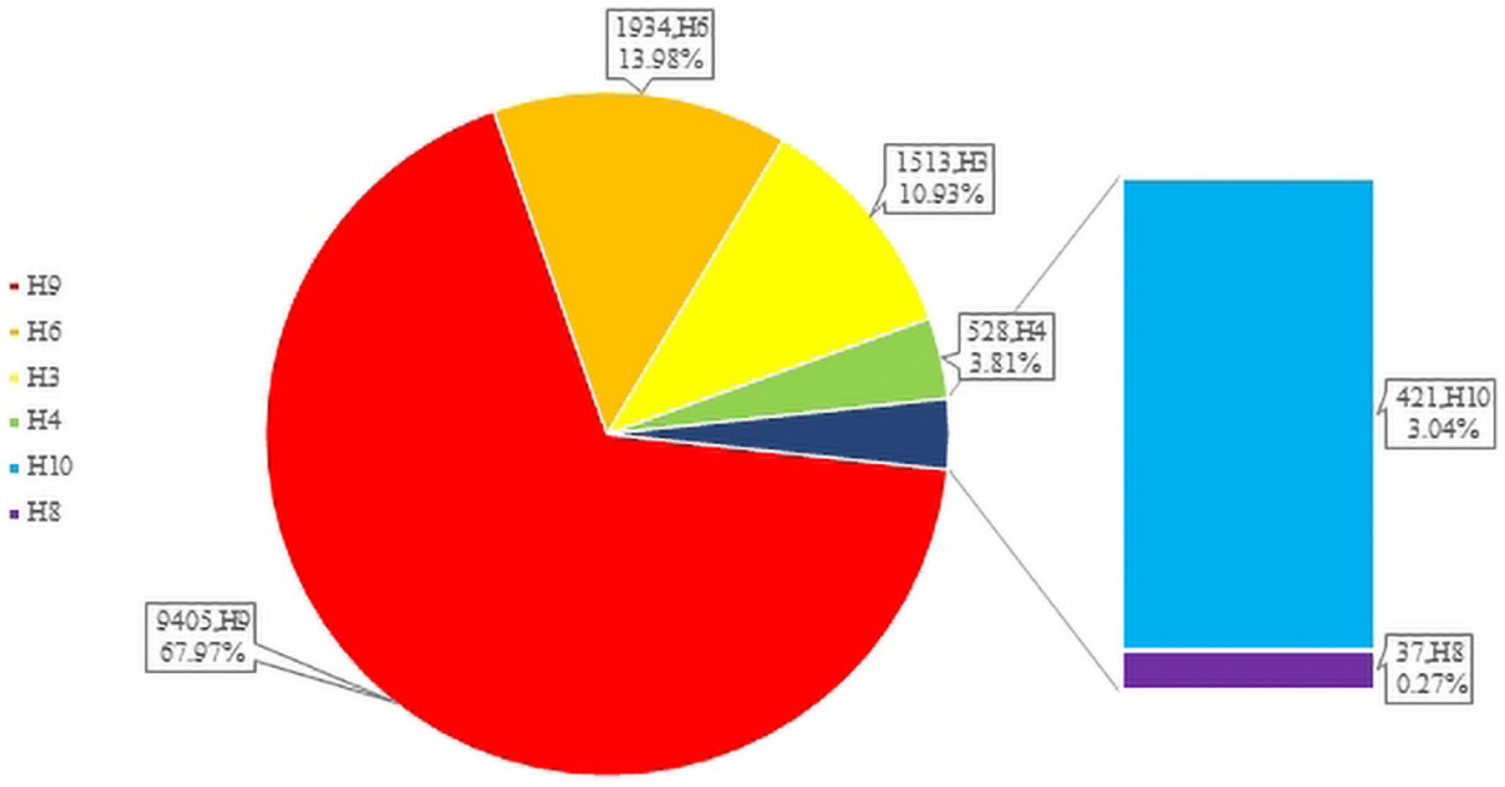
The numbers of LPAIVs in poultry.

**Figure 4:**
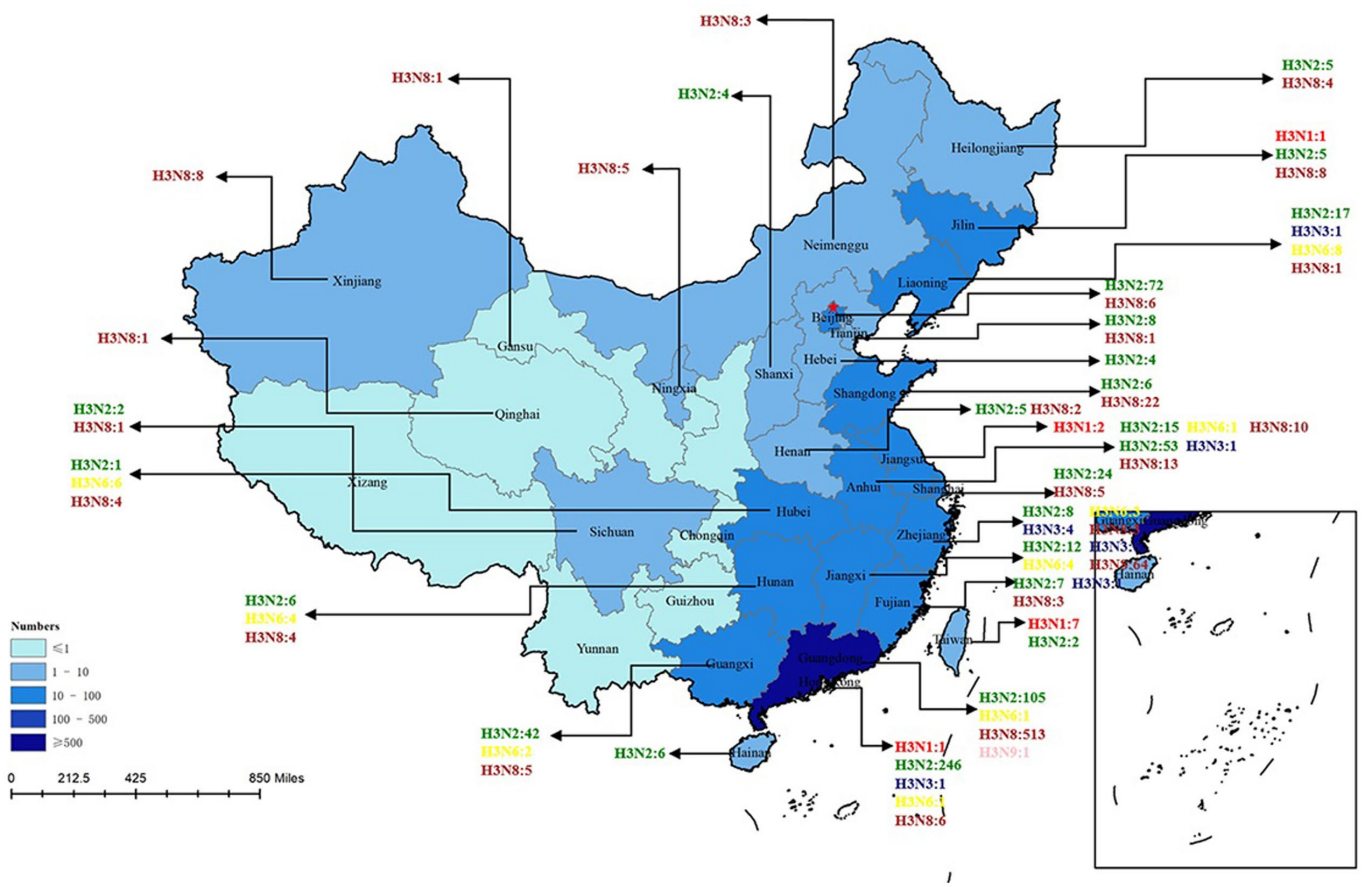
H3 AIV distribution in Chinese provinces. The HA sequences were downloaded from the GISAID and NCBI databases. The provinces where H3-subtype AIVs were isolated are marked with blue, and the darker the blue color is, the more H3-subtype AIVs were isolated in the province. H3N1 is marked in red; H3N2 is marked in green; H3N3 is marked in midnight blue; H3N5 is marked in blue; H3N6 is marked in yellow; H3H8 is marked in brown; and H3N9 is marked in pink. There are 6 H3N? sequences only found in China that are not marked in this figure (N? Indicates unidentified NA subtype).

H3-subtype AIV had the ability to infect various mammalian species, such as swine, canine, feline, equine, and even ferret. In recent years, avian influenza surveillance data have shown that the H3-subtype AIV is one of the main circulating avian influenza subtypes, especially in poultry (with a prevalence of approximately 10.93%, Figure 3). H3-subtype AIV had the highest isolation rate in ducks, at 91.76% (Yang et al., 2021). The H3N2 and H3N8 subtypes are the most important subtypes in poultry in China and are primarily found in southeastern China, roughly the same as the main route of wild bird migration (Qi et al., 2021). Guangdong Province had the highest amount of H3-subtype AIV isolated, with 620 isolates. Shandong, Anhui, Guangxi and other provinces were also found to have a number of H3-subtype AIVs (Figure 4).

**Figure 5:**
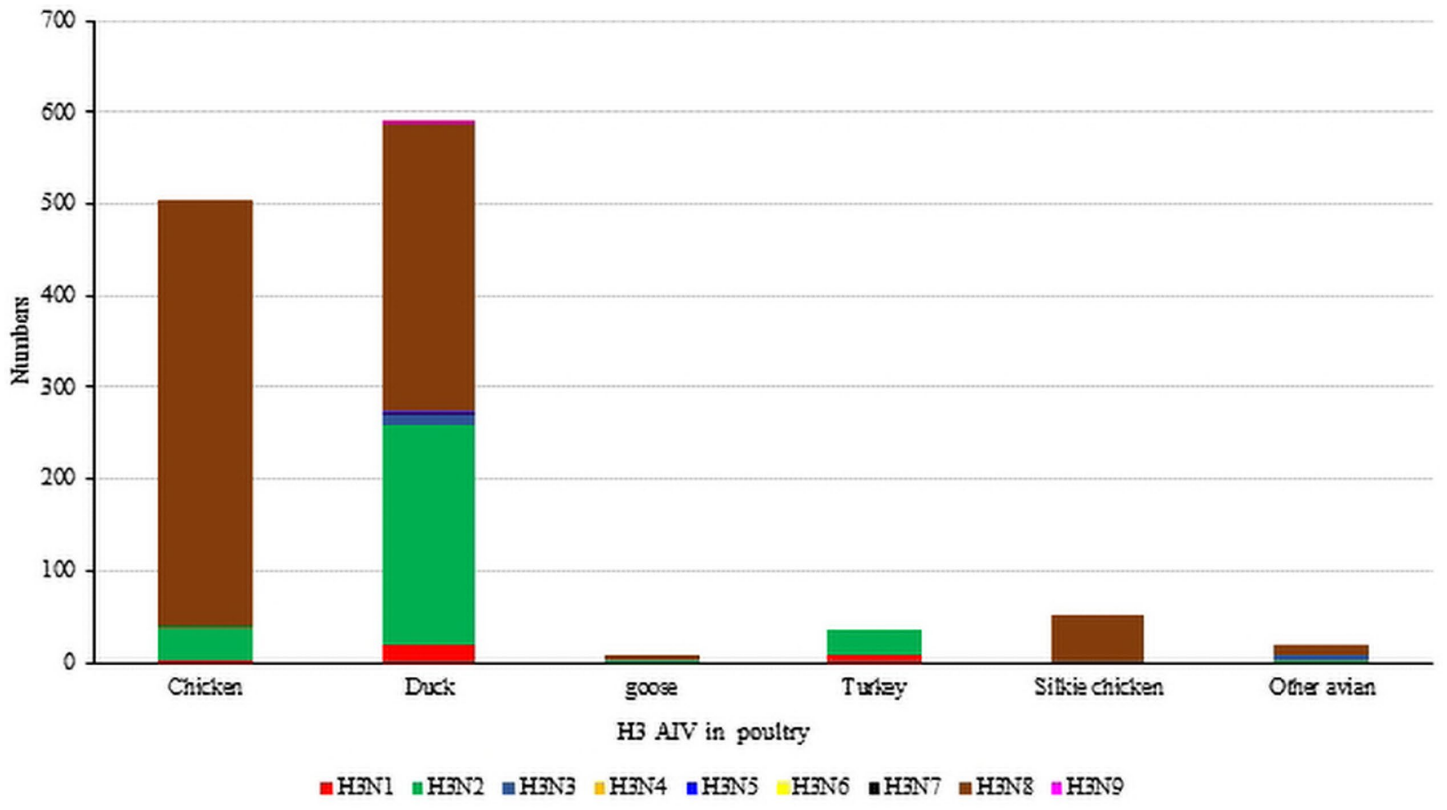
The number of H3 subtypes prevalent in poultry. The HA sequences were downloaded from the GISAID and NCBI databases; H3N? (unidentified NA subtype) sequences are not included here.

Unlike waterfowl, chickens are not the natural hosts of the H3 influenza virus, and chickens infected with H3-subtype AIV are typically symptom-free. However, certain H3-subtype AIV strains have been observed to cause disease in chickens, and recent serological tests on chickens in China confirmed that H3-subtype AIV has spread across a wide geographical area (Pu et al., 2009). It has been proven that live poultry markets (LPMs) are likely the main source of H3-subtype AIV infection in chickens. The conditions of LPMs accelerate the adaptation of AIV to new hosts, and some viruses from wild avian can infect chickens directly without prior adaptation (Bi et al., 2016; Yuan et al., 2016). By July 2023, 503 chicken H3-subtype AIV sequences, 589 duck H3-subtype AIV sequences and 9 goose H3-subtype AIV sequences had been submitted. The prevalence of H3-subtypes AIV in poultry is shown in Figure 5.

### 2.3 Epidemic of H3-subtype avian influenza in wild birds

**Figure 6:**
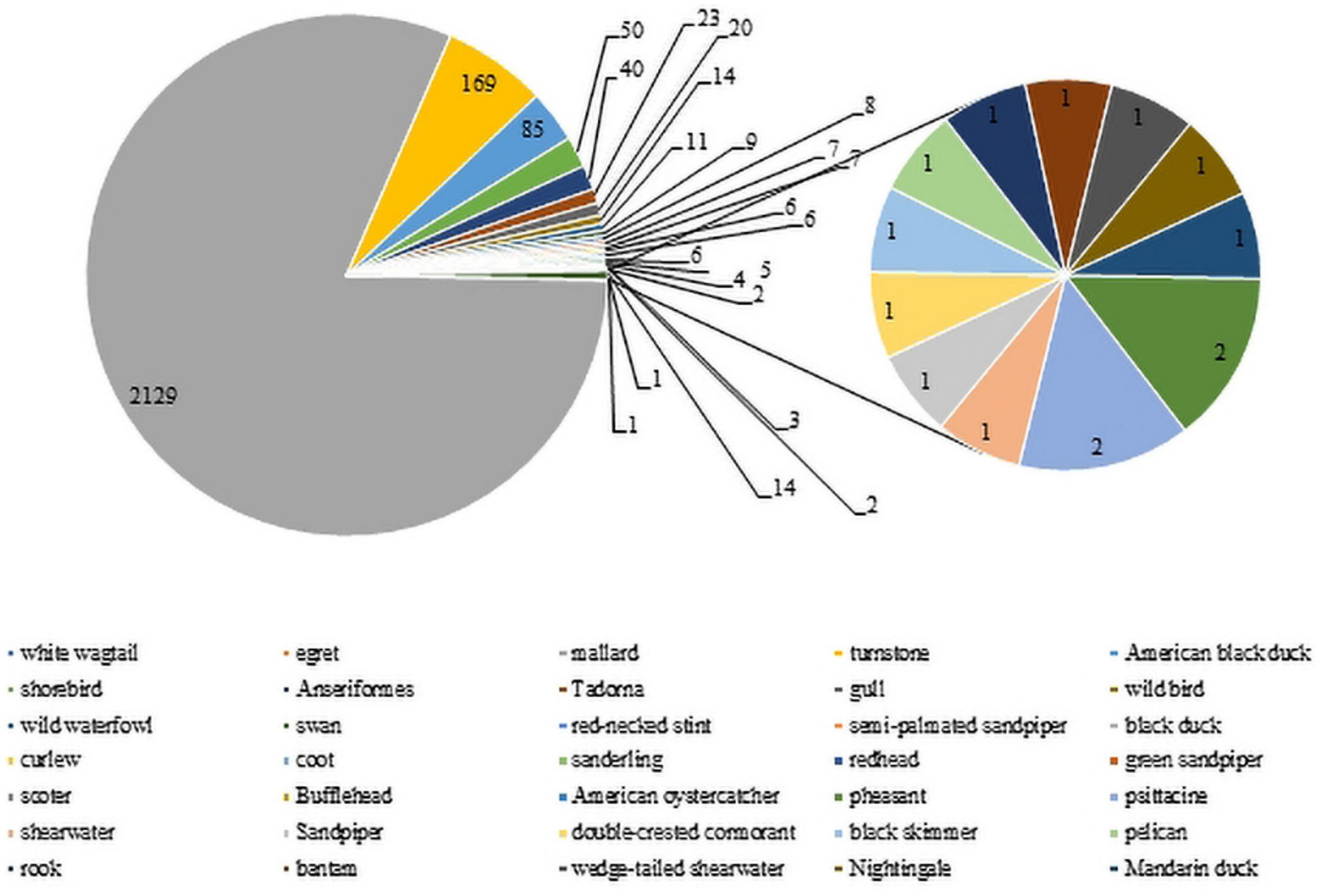
H3 AIV distribution in wild birds. The numbers of H3 virus isolates from different wild birds are shown according to their submission information in the GISAID and NCBI databases.

Wild birds, particularly wild waterfowl, were considered to be natural hosts of AIV (Elbers and Gonzales, 2021). There is evidence that wild bird migration was an important route leading to the evolution and recombination of influenza virus and their spread to poultry and mammals (Mine et al., 2019). To date, H3-subtype AIV has been found in many kinds of wild avian species in several nations and regions. There are 2,763 H3-subtype AIV HA sequences from wild birds in the GISAID and NCBI databases. The main isolation species were mallard duck (2,129 strains), turnstone (169 strains) and American black duck (85 strains), with mallard duck as the main host (81%, Figure 6), and H3N8 was the dominant strain, with 7,396 strains detected. Green-headed ducks have strong resistance to influenza virus and mild symptoms upon infection. The virus can be transmitted through direct contact and the respiratory tract during migration, and its secretions and excreta may also cause the virus to spread (Kaleta et al., 2005).

### 2.4 Epidemic of H3-subtype avian influenza in mammals

**Figure 7:**
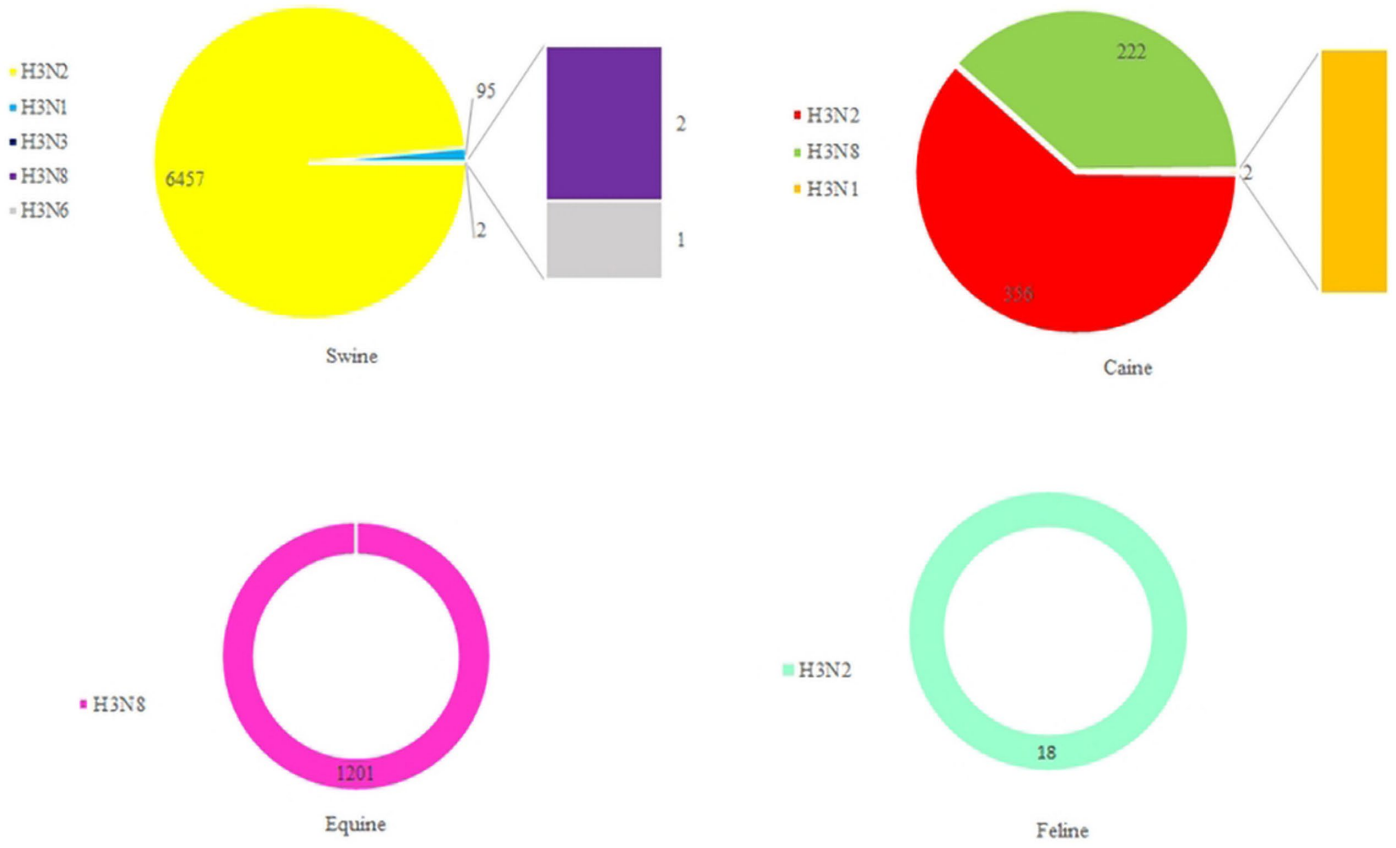
The number of H3 AIVs in mammals. HA sequences were downloaded from the GISAID and NCBI databases.

Based on statistics, it has been determined that the H3-subtype of influenza virus is capable of infecting a diverse range of hosts. These include not only avian hosts but also various mammals, such as swine, canine, equine, and even humans (Figure 7). Stable mammalian transmission capacity is one of the prerequisites for the spread of the new influenza virus in the population (Yun et al., 2004; Pu et al., 2009; Tu et al., 2009; Choi et al., 2012; Yondon et al., 2014). In March 1989, Guo et al. (1992) isolated an avian-origin H3N8 subtype equine influenza virus from horses with severe respiratory disease in northeast China. In 1998, Zhou et al. detected a triple recombinant H3N2 influenza A virus containing genes from human, classical swine, and avian influenza viruses in North American swine (Zhou et al., 1999). Since 2005, there have also been frequent incidents of canine infection with avian-origin H3N2 influenza viruses in South Korea and China (Said et al., 2011; Yang et al., 2014). During September to December 2011, 162 spotted seals in New England died after contracting H3N8 subtype AIV, which resulted in severe pneumonia (Anthony et al., 2012). A study by Guan et al. in 2019 revealed that the H3N2 subtype of AIV isolated from an LPM in China had the ability to infect mice, replicate efficiently in mice, bind to avian- and human-type receptors, and achieve interspecies respiratory droplet transmission in guinea pigs and ferrets (Guan et al., 2019). Between April 2022 and March 2023, there were three cases of human infection with AIV subtype H3N8 in China with severe respiratory symptoms, and all three human cases were associated with exposure to live poultry (WHO, n.d.; Bao et al., 2022; Cheng et al., 2022; Yang et al., 2022). All these lines of evidence of H3-subtype AIV infecting mammals indicate their superb cross-species transmissibility, which is an enormous problem to farming industry and human health and safety.

## 3 Cross-species transmission

**Figure 8:**
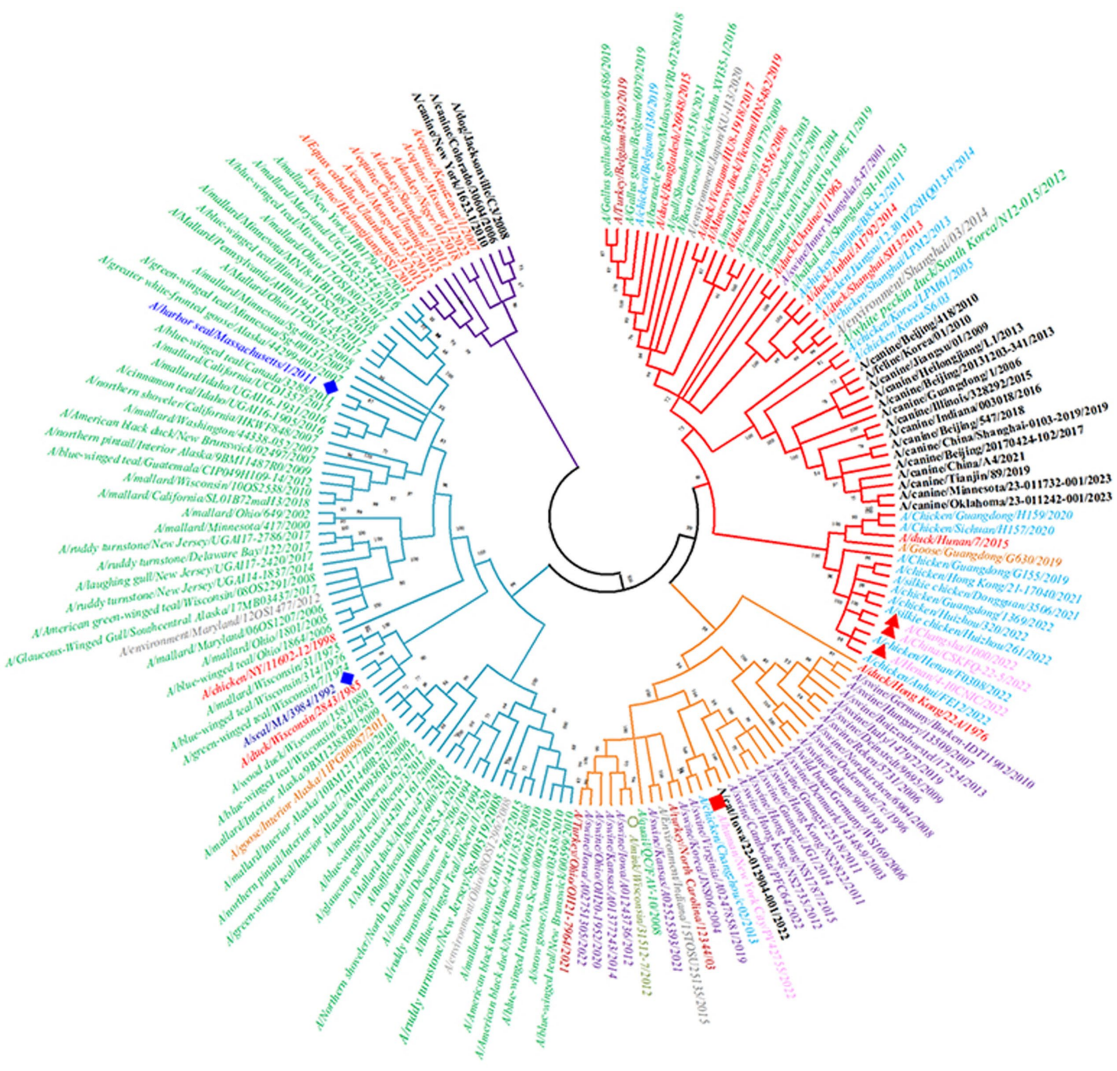
Phylogenetic tree of the HA genes of 19 H3-subtype AIVs. The red branches of the phylogenetic tree represent the Eurasian avian lineage, the light blue branches represent the North American avian lineage, the orange branches represent human/swine lineages, and the purple branches represent equine lineages. Second, the leaves of the phylogenetic tree are red for ducks, green for wild birds, light blue for chickens, brown–red for turkeys, purple for pigs, black for canines and felines, orange for geese, pink for humans (where avian-derived human influenza viruses are marked with red triangles and seasonal human influenza viruses are marked with red squares), and dark blue (diamonds) for seals.

There are differences in the antigenicity of H3-subtype influenza viruses in different hosts, but according to previous reports, the phenomenon of cross-species transmission also occurs occasionally. There are two major lineages of H3-subtype AIV based on phylogenetic analysis and geographical location: the Eurasian lineage and the North American lineage, which are deeply divided into the human/swine lineage and the avian and equine lineage (Figure 8). Studies have shown that both avian H3 lineages can cross-species to infect swine and canines, however, the Eurasian lineage is considered to have broader range of hosts, such as pigs, horses, dogs, ferrets, seals and humans (Liu et al., 2009).

In wild waterfowl, the detection rate of H3-subtype AIV is very high. Avian influenza virus can also spread across continents through wildfowl migration. H3N2 Hong Kong influenza virus recombined with human influenza H2N2 polygenes in 1968, which caused the Hong Kong human influenza pandemic, and the HA of H3N2 AIV deviated from that of the avian-origin AIV. Therefore, the antigenicity of contemporary human H3 influenza virus is significantly different from that of H3 AIV (Liu et al., 2009). H3-subtype AIV was introduced into horses in the 1960s, and its HA antigenicity has changed since then, gradually leading to the formation of a separate lineage (Woodward et al., 2015). In 2006, H3N2 AIV entered the canine population and formed a lineage of canine influenza virus. The haemagglutinin of all porcine H3 viruses are derived from those of human viruses. Therefore, this lineage is in the same lineage as human influenza virus in the evolutionary tree. Notably, in 2022, there were three cases of human infection with avian-derived H3N8 influenza virus in China. We downloaded their HA protein-encoding gene sequences from the NCBI, which were A/Changsha/1000/2022, A/China/CSKFQ-22-5/2022 and A/Henan/4-10CNIC/2022. After alignment and pairwise comparison with other H3-subtype AIV sequences, the HA of human H3N8 strain A/Henan/4-10CNIC/2022 and chicken H3N8 strain A/chicken/Henan/F0308/2022 had the highest homology, indicating that the new H3N8 virus causing human infection was derived from chickens (Yang et al., 2022). The above studies show that H3-subtype AIV still has the risk of causing a pandemic in the human population.

## 4 Characteristics of the viral genome

### 4.1 Selection analysis

**Figure 9:**
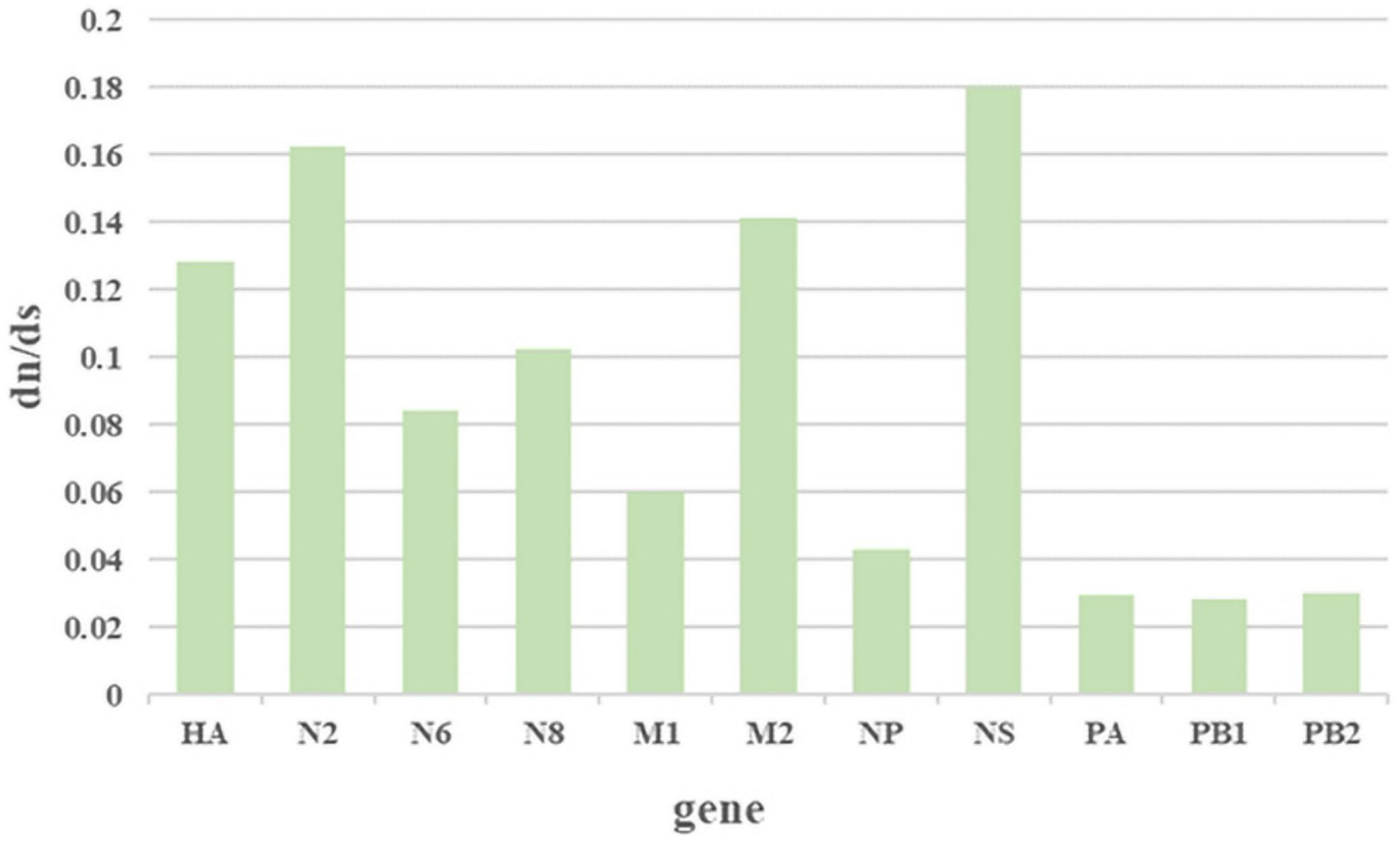
The results of the selection analysis. The associated amino acid changes were analyzed using MEGA 11. Consensus sequences were aligned, and mutations were recorded. The positions of the mutations for each enzootic cluster were confirmed manually. The number of amino acid changes in each enzootic cluster was counted.

The ratio of nonsynonymous substitutions to synonymous substitutions (dN/dS) is commonly used to quantify the magnitude of evolutionary selection pressure on protein-coding genes. A higher dN/dS ratio reflects the potential influence of positive selection on genes during evolution, contributing to adaptation to environmental changes or the acquisition of new functions. In this study, H3NX AIV sequences were aligned and screened using MEGA 11.0 software. The dN/dS ratio was evaluated for each segment of the H3NX AIV using Launch DnaSP6 software. Specifically, all sequences were selected without annexed bases and had complete coding regions. The analysis that indicated that the NS, NA-N2, HA, and M2protein gene segments may have experienced higher selection pressure than the others (Figure 9; Table 1), which exhibited higher dN/dS substitution ratios.

**Table 1:** The result of the dN/dS calculation.

Mean dN/dS	dN	dS	dN/dS
HA	0.06379	0.4966	0.128453
NA-N2	0.05904	0.36369	0.162336
NA-N6	0.04017	0.47573	0.084439
NA-N8	0.05026	0.48927	0.102724
M1	0.01635	0.2705	0.060444
M2	0.03018	0.21376	0.141186
NP	0.02075	0.48022	0.043209
NS	0.08151	0.45208	0.1803
PA	0.01253	0.42086	0.29772
PB1	0.01242	0.43912	0.028284
PB2	0.01277	0.42527	0.030028

### 4.2 Three-dimensional structure of H3-subtype AIV HA protein

Studies have shown that mutations in the HA protein receptor binding domains Q226L and G228S will change the binding of viral receptors so that they can bind not only to α-2,3-linked SA but also to α-2,6-linked SA (Matrosovich et al., 2000). Then, human infection with avian influenza may be possible. In addition, the substitution of R62I, N92, A144G and N145S in H3-subtype AIV HA protein may also increase the binding ability of the virus to human receptors (West et al., 2021).

**Figure 10:**
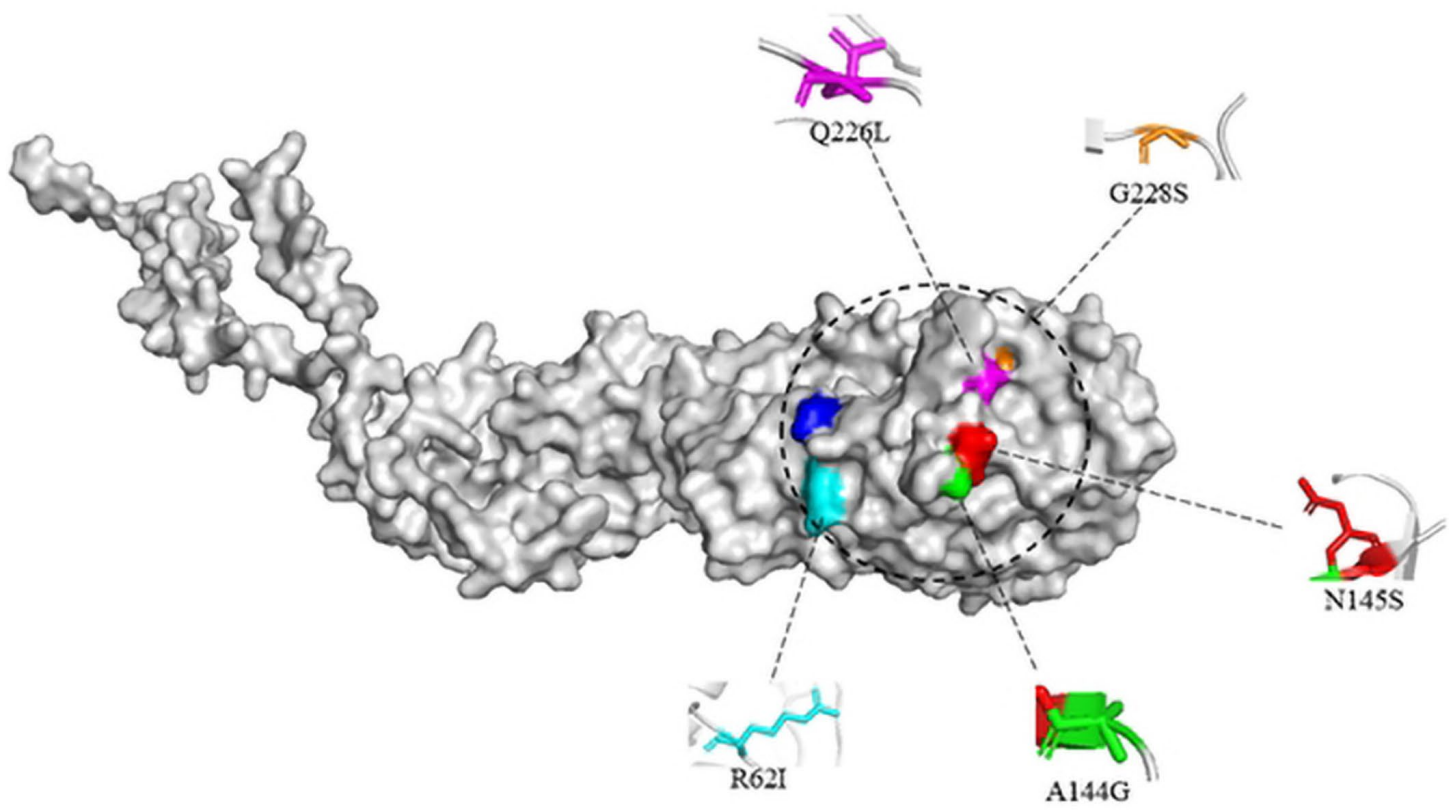
Structural simulation of the H3 avian influenza virus HA protein. The structure of the HA monomer is shown, and some key amino acid sites affecting the viral biological properties are labelled. The structure was homology modeled with the HA protein templates of A/chicken/Hong Kong/37/1978(H3N2) and A/Hong Kong/1/1968 (H3N2) viruses and presented with PyMOL software.

Here, the three-dimensional structure of the H3-subtype AIV HA protein was built by PyMOL software. A/chicken/Hong Kong/37/1978 (H3N2) and A/Hong Kong/1/1968 (H3N2) were selected for structural prediction. The structure of H3-subtype AIV HA protein was built through homology modeling, and suitable templates were selected using Alphafold2 (Figure 10). Only the monomeric structure of HA is displayed in the image, with key amino acid residues that affect viral biological characteristics labelled.

## 5 Prevention and control of H3-subtype AIV

The transmission of H3-subtype avian influenza virus from wild birds to poultry is characterized by unpredictability and uncontrollability. Therefore, at this stage, vaccination remains the best measure to prevent and control outbreaks of H3-subtype avian influenza. However, current research on AIV vaccines is mostly focused on the H5, H7, and H9 subtypes, with a lack of vaccines specifically targeting H3-subtype avian influenza on the market. Nonetheless, H3-subtype influenza virus-like particle vaccine (Lee et al., 2013; Schwartzman et al., 2015; Mai et al., 2023), epitope hemagglutinin vaccine (Bullard et al., 2021; Allen and Ross, 2022), and mucosal vaccine (Wang et al., 2020) have made some progress in humans and pigs, which could serve as a foundation for the future development of safer, broader spectrum, and more immunoprotective H3-subtype avian influenza virus vaccine.

Additionally, H3-subtype AIV, as a LPAIV, is more easily overlooked. Treatment after illness is not worthwhile. Isolating the source of infection is an effective measure for avoiding H3-subtype AIV infection in poultry. Therefore, it is particularly important for the prevention and control of H3-subtype AIV to strengthen the regular census and monitoring of H3-subtype AIV in wild birds and poultry.

## 6 Conclusion

H3-subtype AIV is in a state of evolution and recombination. Frequent recombination occurred between different subtypes, and cross-species transmission occurred. Moreover, multiple mammalian adaptive mutation sites were found on H3-subtype AIV HA protein. These findings indicate that H3-subtype AIV is gradually adapting to mammals and even humans. Therefore, the monitoring of mutation and recombination in H3-subtype AIVs should be continued, and efficient vaccines should be developed to prevent and control the prevalence of H3-subtype AIV.

## Author contributions

JY: Writing – original draft. QY: Writing – review & editing. JL: Data curation, Software, Writing – review & editing. YZ: Writing – review & editing. MH: Writing – review & editing. YG: Funding acquisition, Project administration, Writing – review & editing.
